# Lumbar puncture opening pressure, brain network hub integrity, and delirium in herpes simplex virus encephalitis: a prospective cohort study

**DOI:** 10.3389/fneur.2026.1837032

**Published:** 2026-06-18

**Authors:** Yandong Sun, Ye Ding, Bin Wang, Xingcheng Duan, Shouyang Zhu, Shengtao He, Jueyue Yan, Jingchen Zhang

**Affiliations:** 1 Emergency Department of People’s Hospital of Anji, Huzhou, Zhejiang, China; 2 Department of Neurology, QuZhou KeCheng People‘s Hospital, Quzhou, Zhejiang, China; 3 Quzhou Renyi Traditional Chinese Medicine Hospital Co., Ltd., Quzhou, China; 4 Department of Critical Care Medicine, The First Affiliated Hospital, Zhejiang University School of Medicine, Hangzhou, Zhejiang, China

**Keywords:** degree centrality, delirium, functional MRI, HSV encephalitis, intracranial pressure

## Abstract

**Background:**

Herpes simplex virus (HSV) encephalitis is the most common sporadic viral encephalitis, frequently complicated by delirium. Intracranial pressure (ICP) elevation is a common feature, but its role in delirium via brain functional network disruption remains unclear. This study investigated whether surrogate marker of ICP burden (LP opening pressure) mediates delirium in HSV encephalitis by reducing degree centrality (DC), a measure of functional network hub integrity, in key brain regions.

**Methods:**

In a prospective two-center cohort study (December 2023–November 2025), 55 HSV encephalitis patients and 30 healthy controls underwent clinical assessments, ICP monitoring, and resting-state functional MRI (rs-fMRI). Delirium was evaluated using the Delirium Rating Scale (DRS; ≥12 indicating presence). DC was calculated from rs-fMRI data in regions of interest. Associations were analyzed via logistic regression, ANOVA, linear regression, and mediation models with bootstrapping.

**Results:**

Of 55 patients, 22 (40%) developed delirium. The delirium group had higher LP opening pressure (23.5 ± 4.8 vs. 15.5 ± 3.2 cmH₂O; *p* < 0.001), lower Glasgow Coma Scale scores, and worse outcomes. Elevated LP opening pressure (≥20 cmH₂O) increased delirium odds (OR 1.86; 95% CI 1.58–2.11; *p* < 0.001), amplified in subgroups with severe inflammation. DC was reduced in the delirium group in the right amygdala, right hippocampus, left insula, and left precuneus (all *p* < 0.05 vs. non-delirium and controls). LP opening pressure inversely correlated with DC in these regions (except left insula), and DC inversely correlated with DRS scores. Mediation analyses confirmed partial mediation by DC in the right amygdala (*β* = 0.129; 95% CI 0.079–0.158; *p* = 0.031), right hippocampus (*β* = 0.312; 95% CI 0.277–0.458; *p* = 0.002), and left precuneus (*β* = 0.275; 95% CI 0.187–0.398; *p* = 0.011).

**Conclusion:**

Our exploratory findings suggest that LP opening pressure may be partially associated with delirium in HSV encephalitis through reduced degree centrality in limbic and default mode network hubs. As a preliminary investigation, these hypothesis-generating results highlight ICP management and DC as potential imaging biomarkers that warrant further validation in larger, longitudinal studies for delirium risk stratification and prevention.

## Introduction

Herpes simplex virus (HSV) encephalitis is a severe acute viral infection of the central nervous system, primarily caused by HSV-1, and represents the most common sporadic viral encephalitis in adults worldwide (1). With an annual incidence of 2–4 cases per 100,000 population, untreated HSV encephalitis carries a mortality rate of up to 70%, while even with antiviral therapy such as acyclovir, survivors often face significant neurological sequelae, including cognitive deficits and epilepsy (2). Delirium emerges as a frequent and debilitating complication during the acute phase of HSV encephalitis, affecting up to 20–60% of patients and manifesting as fluctuating attention, altered consciousness, and cognitive disturbances (3). In neurointensive care settings, delirium is associated with prolonged hospital stays, increased mortality, and heightened risk of long-term cognitive impairments (4). Current management strategies for delirium in HSV encephalitis remain largely symptomatic, emphasizing supportive care without mechanism-based preventive approaches, highlighting the urgent clinical need for research into its underlying pathophysiology to enable targeted interventions.

Existing knowledge on delirium in HSV encephalitis identifies several risk factors, including advanced age and systemic inflammation markers such as elevated C-reactive protein (5). Notably, brain edema and intracranial pressure (ICP) elevation are common pathophysiological processes in HSV encephalitis, driven by viral-induced inflammation and often leading to cerebral herniation if unmanaged (6). ICP dysregulation has been linked to consciousness disorders in various neurological conditions, where sustained elevations impair cerebral perfusion and exacerbate neuronal injury (7). However, the specific association between ICP elevation and delirium in HSV encephalitis, along with its mechanistic underpinnings, remains underexplored. Contemporary theories of delirium posit that its core pathophysiology involves acute disruptions in large-scale brain functional networks, particularly the default mode network and limbic–cortical systems, which are essential for attention, emotion regulation, and consciousness (8). Degree centrality (DC), a key graph theory metric from resting-state functional magnetic resonance imaging (fMRI), quantifies the importance of brain regions as functional network hubs by measuring their connectivity strength (9). Alterations in DC, indicative of hub dysfunction, have been observed in neuropsychiatric disorders, including reduced DC in regions like the amygdala and hippocampus during states of cognitive vulnerability (10). Despite these insights, a critical knowledge gap persists: no studies have investigated whether ICP elevation in HSV encephalitis mediates delirium occurrence by impairing functional network hubs (i.e., reducing DC) in specific brain regions. This constitutes the central scientific question addressed by the present study.

We therefore aimed to investigate the interrelationship between ICP, functional hub connectivity, and delirium in HSV encephalitis. We hypothesized that elevated LP opening pressure constitutes a key risk factor for delirium, and that this effect is, at least in part, mediated by impaired functional integration of specific brain hubs—particularly within the limbic system and default mode network—as measured by decreased degree centrality on resting-state fMRI.

To this end, we prospectively collected clinical data, ICP monitoring results, and resting-state fMRI scans from HSV encephalitis patients. We first compared baseline characteristics across groups, then analyzed correlations between brain region DC values, ICP, and delirium scores, and finally employed mediation analysis models to confirm the mediating effects of DC. This study provides the first insights into HSV encephalitis-associated delirium from the perspective of functional network hub dysfunction, potentially informing the development of imaging-based biomarkers for targeted interventions.

## Method

This two-center cohort study was conducted at the First Affiliated Hospital, Zhejiang University School of Medicine, and the Quzhou Kecheng People’s Hospital, China, between December 2023 and November 2025. Data from patients who received a clinical diagnosis of encephalitis (International Classification of Diseases, Ninth Revision code 054.3) and had a positive cerebrospinal fluid (CSF) polymerase chain reaction test for HSV DNA data were prospectively collected and analyzed. All included patients underwent neuroimaging. The study protocol was approved by the Ethics Committees of both participating hospitals (Quzhou Kecheng People’s Hospital and the First Affiliated Hospital, Zhejiang University School of Medicine; approval numbers: KCYY2025-K53 and IIT20230253B-R2) and was conducted in accordance with the principles of the Declaration of Helsinki. The reporting of this study follows the Strengthening the Reporting of Observational Studies in Epidemiology (STROBE) guideline.

### HSE patients

Our inclusion criteria were as follows: admission to the Intensive Care Unit (ICU) or Department of Neurology with a length of stay of 24 h or more, a diagnosis of possible acute encephalitis, and a CSF polymerase chain reaction test positive for HSV DNA during hospitalization. The diagnosis of possible acute encephalitis was made in accordance with international guidelines (11) and required the presence of acute changes in mental status or behavior lasting 24 h or longer, accompanied by at least two of the following: fever within 72 h before or after presentation, generalized or partial seizures, new focal neurological deficits, CSF white blood cell count ≥5 cells/μL, or neuroimaging/electroencephalogram findings suggestive of encephalitis. Exclusion criteria were: neuroimaging not performed or performed >14 days after ICU admission, pre-existing neurological conditions that could confound cerebral MRI analysis (e.g., brain tumor or severe traumatic brain injury), poor-quality MRI, and incomplete clinical information.

### Lumbar puncture and intracranial pressure measurement

Following admission and after exclusion of contraindications (e.g., coagulopathy, local infection, or signs of significant mass effect on neuroimaging), patients underwent a diagnostic lumbar puncture. CSF pressure was measured using direct manometry at the time of the procedure, with the recorded value in cmH_2_O representing the ICP at that time point. Results from the initial diagnostic lumbar puncture, including CSF white blood cell count, protein, and glucose levels, were collected as baseline laboratory data.

For clinical management, some patients required repeated lumbar punctures for therapeutic drainage or monitoring. In all cases, the surrogate marker of ICP burden (LP opening pressure) was defined as the single highest CSF pressure value (in cmH_2_O) obtained from any lumbar puncture performed during the acute hospitalization period. This peak value was used for all subsequent analyses correlating ICP with clinical and neuroimaging outcomes.

Lumbar puncture opening pressure measured by direct manometry is a well-established, non-invasive surrogate for intracranial pressure in patients with suspected viral encephalitis when continuous invasive monitoring is not clinically indicated (11). In the present study, all patients underwent an initial diagnostic LP after neuroimaging ruled out contraindications. Additional therapeutic or monitoring LPs were performed as clinically indicated for CSF drainage or pressure management. LP opening pressure was defined as the highest CSF opening pressure recorded from any LP during the acute hospitalization period. This approach captures the maximum pressure exposure and aligns with clinical practice in HSE, where invasive intracranial pressure monitoring is typically reserved for patients with severe cerebral edema or signs of herniation.

### Clinical information

Patient history, along with clinical, laboratory, and neuroimaging data, were collected from medical records. A neuropsychological examination was performed at ICU admission. Delirium was assessed twice daily by trained research staff using the Delirium Rating Scale (DRS) from admission until ICU discharge or clinical stabilization; a DRS score of ≥12 was used to define the presence of delirium, with higher scores indicating greater severity. Mental status at ICU admission was evaluated using the GCS (range 3–15), where a score of less than 8 corresponds to coma.

Healthy control participants were recruited from individuals attending the affiliated hospitals for routine health examinations. To be eligible as a control, an individual must have reported no history of neurological disorder, have undergone a brain MRI that showed no structural abnormalities, and have completed a standard clinical assessment. For all controls, detailed medical histories were recorded, including information on lifestyle, vascular risk factors, and relevant clinical parameters.

### Outcome assessment

Functional outcomes were evaluated 90 days after ICU admission using the Modified Rankin Scale (mRS) (12, 13). The mRS is a 7-level ordinal scale ranging from 0 (no symptoms) to 6 (death), which assesses the degree of disability or dependence in daily activities following neurological injury; a lower score indicates a better functional status. Assessments were conducted systematically based on available follow-up consultation records and/or information provided by treating physicians. A good functional outcome was defined as an mRS score of 0–2, denoting no symptoms to slight disability. Patients discharged home with functional independence prior to the 90-day timepoint were also classified as having a good outcome. Conversely, a poor functional outcome was defined as an mRS score of 3–6, indicating moderate to severe disability or death.

### Neuroimaging examination

Brain MRI, including resting-state functional MRI, was performed within the first week following ICU admission whenever the patient’s clinical condition permitted the examination. All scans were acquired using a Siemens 3 Tesla Trio Tim MRI system equipped with a 32-channel head coil. For functional imaging, blood-oxygen-level-dependent (BOLD) signals were obtained with a gradient-echo echo-planar imaging sequence using the following parameters: repetition time (TR) = 2000 ms, echo time (TE) = 30 ms, flip angle = 90°, field of view (FOV) = 200 × 200 mm^2^, matrix size = 64 × 64, 33 axial slices with a slice thickness of 4.8 mm and no gap, yielding 200 volumes per run. For anatomical reference and spatial normalization, high-resolution three-dimensional T1-weighted structural images were acquired for each participant using a magnetization-prepared rapid gradient echo sequence (MPRAGE) with the following parameters: TR = 1900 ms, TE = 2.48 ms, inversion time (TI) = 900 ms, flip angle = 9°, FOV = 256 × 256 mm^2^, and an isotropic voxel size of 1 × 1 × 1 mm^3^ encompassing 128 sagittal slices.

### Data processing

All preprocessing of resting-state functional MRI (rsfMRI) data was carried out using Statistical Parametric Mapping version 12 (SPM12; Wellcome Centre for Human Neuroimaging, London, UK) and the Data Processing and Analysis of Brain Imaging toolbox. The standard pipeline included the following steps: removal of the initial volumes to allow for signal equilibration, slice timing correction to account for acquisition delays, and realignment of all volumes to the first image for head motion correction. The functional images were then co-registered to each participant’s high-resolution T1-weighted anatomical scan. Subsequent spatial normalization to the standard Montreal Neurological Institute (MNI) template was performed using the DARTEL algorithm, and the normalized images were resampled to an isotropic voxel size of 3 mm. Spatial smoothing was applied with a 6-mm full-width at half-maximum Gaussian kernel. Finally, to mitigate the effects of low-frequency drift and high-frequency physiological noise, a temporal band-pass filter (0.01–0.08 Hz) was applied to the time series of each voxel, and linear detrending was performed.

### Calculation of DC

Voxel-wise DC across the whole brain was calculated from the preprocessed fMRI data according to established methodologies (14). Initially, a whole-brain gray matter mask was applied. For each individual, Pearson’s correlation coefficients were computed between the time series of every pair of voxels within the mask. The resulting correlation coefficients were then converted to normally distributed *Z*-scores using Fisher’s *r*-to-*z* transformation. Subsequently, an individual-level functional connectivity network was constructed by applying a correlation threshold of *r* > 0.25 to the *z*-scored matrices, as reported previously (15). To evaluate the robustness of our findings, dataset-specific sensitivity analyses were performed using alternative thresholds (*r* > 0.2 and *r* > 0.3) as well as a weighted network approach (retaining edge weights without binarization). Negative correlations were excluded from DC calculations to focus on positive functional connectivity and maintain consistency with the interpretability of degree centrality in clinical neuroimaging studies; the biological relevance of anti-correlated networks is increasingly recognized but remains debated, particularly in the context of global signal regression preprocessing. The DC value for a given voxel was defined as the count of its connections that survived this threshold. Finally, to allow for group-level comparison, the voxel-wise DC maps for each subject were transformed into *z*-score maps. All voxel-wise and ROI-based analyses additionally included mean FD and scrubbed volume count as covariates to further minimize motion-related bias.

To identify brain regions with significant DC differences between HSE patients and healthy controls, a two-sample *t*-test was performed on the *z*-scored DC maps using the REST software. Correction for multiple comparisons was carried out using Monte Carlo simulations as implemented in the AlphaSim program (within AFNI). This method estimates the probability of cluster occurrence by chance for a given per-voxel threshold. The statistical significance threshold was set at a voxel-level height of *p* < 0.05combined with a minimum cluster size of 198 voxels, corresponding to a family-wise error (FWE) corrected *p* < 0.05. The simulations were confined to the gray matter mask.

### Statistical analysis

Statistical analyses were performed using R software (version 4.4.3). Continuous variables are presented as mean ± standard deviation or median (interquartile range) as appropriate, and categorical variables as number (%).

To assess the association between elevated intracranial pressure (ICP; defined as lumbar puncture opening pressure ≥20 cmH₂O) and delirium, logistic regression was employed to calculate odds ratios (ORs) with 95% confidence intervals (CIs). Given the relatively small cohort size, we utilized parsimonious multivariable models including only prespecified, clinically relevant covariates (e.g., age, sex, and key inflammatory markers) to minimize overfitting. Model assumptions were verified through residual and Q–Q plot inspections, and multicollinearity was assessed using variance inflation factors (VIF < 5 for all predictors). Influential observations were identified using Cook’s distance (>1), and sensitivity analyses were conducted by excluding these cases. Internal validation of regression models was performed using 5,000 bootstrap resamples with bias-corrected accelerated (BCa) CIs. For mediation analyses, 5,000 bootstrap replications were used to estimate robust bias-corrected CIs for indirect effects.

Group comparisons for baseline characteristics were conducted using independent samples *t*-tests, Mann–Whitney *U* tests, or Chi-square tests, as appropriate. Differences in DC values across the three groups (Healthy Controls, Non-delirium, Delirium) for the four prespecified regions of interest (ROIs) were assessed using one-way ANOVA, followed by post-hoc pairwise comparisons with Bonferroni correction. For whole-brain voxel-wise analyses, correction for multiple comparisons was performed using Monte Carlo simulation (AlphaSim). All statistical tests were two-sided, and *p* < 0.05 was considered statistically significant. To address potential residual confounding, we performed additional sensitivity analyses adjusting for key ICU-related variables (mechanical ventilation status [yes/no], documented seizure occurrence during hospitalization [yes/no], use of antiseizure medications, and time from symptom onset to antiviral therapy initiation [days]). These covariates were added individually or in small combinations to the parsimonious models to avoid overfitting given the modest sample size. To control for multiple comparisons across the primary regression models, mediation analyses, and four prespecified ROI comparisons, we applied Bonferroni correction (adjusted *α* = 0.05/12 = 0.0042) in addition to the voxel-wise AlphaSim correction already used for whole-brain analyses. Sensitivity analyses using false discovery rate (FDR) correction were also performed and yielded consistent results.

## Results

**Figure 1:**
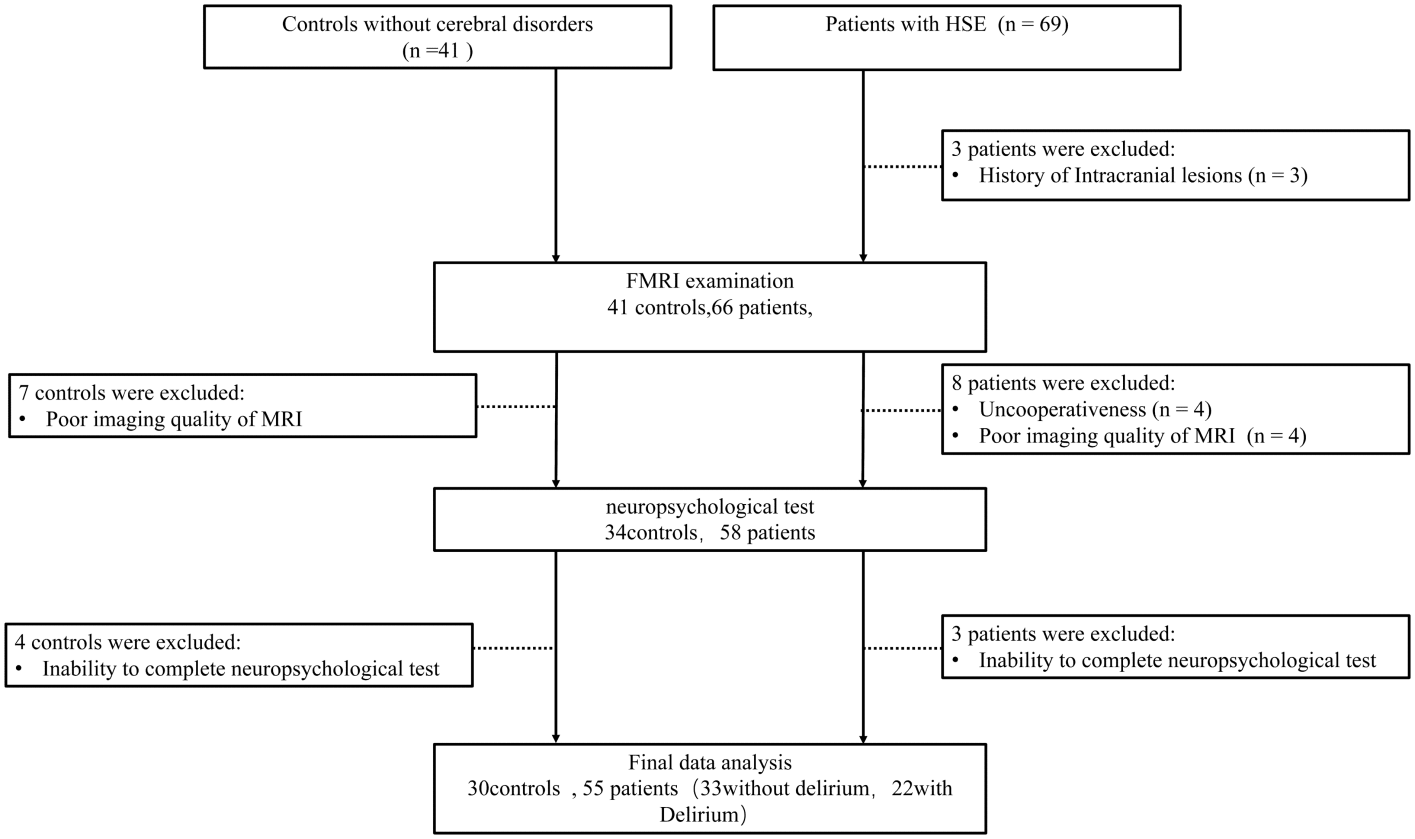
Flow chart of the study.

A total of 69 consecutive patients with confirmed HSV encephalitis and 41 age- and sex-matched healthy controls were prospectively enrolled. The participant flow is illustrated in Figure 1. Prior to neuroimaging, three HSV encephalitis patients were excluded due to a prior history of intracranial lesions. The remaining 66 patients and 41 controls underwent rs-fMRI. Subsequently, seven controls were excluded for poor MRI quality, and eight patients were excluded (four for uncooperativeness during scanning and four for poor image quality), yielding 58 patients and 34 controls eligible for neuropsychological evaluation. An additional four controls and three patients were excluded owing to inability to complete the assessment battery. Thus, the final analytical cohort consisted of 55 HSV encephalitis patients and 30 healthy controls. Of the patients, 22 (40.0%) developed delirium during intensive care unit admission (Delirium group), whereas 33 (60.0%) did not (Non-delirium group).

Baseline characteristics across the three groups are presented in Table 1. No significant differences were observed in age, sex, or body mass index (all *p* > 0.05). However, marked clinical distinctions emerged between the patient subgroups. Upon admission, the Delirium group exhibited lower GCS scores (mean ± SD: 11.8 ± 2.3 vs. 13.5 ± 1.6; *p* < 0.001) and higher baseline DRS scores (17.8 ± 5.4 vs. 8.5 ± 3.2; *p* < 0.001) relative to the Non-delirium group. Diabetes history was more common in the Delirium group (22.7% vs. 12.1%; *p* = 0.02). Moreover, this group experienced prolonged hospital stays (median [IQR]: 22.5 [15.0–30.0] vs. 12.0 [8.0–18.0] days; *p* < 0.01) and poorer 3-month functional outcomes, as evidenced by elevated modified Rankin Scale (mRS) scores (3.5 ± 1.6 vs. 1.8 ± 1.2; *p* < 0.001).

**Table 1:** Baseline characteristics of healthy controls and HSV encephalitis patients stratified by delirium status.

Variable	Healthy controls (*n* = 30)	HSV without delirium (*n* = 33)	HSV with delirium (*n* = 22)	*P*-value
Demographic characteristics				
Age (years, mean ± SD)	42.5 ± 11.8	45.3 ± 12.6	49.7 ± 13.5	0.15
Sex [male, *n* (%)]	14 (46.7)	18 (54.5)	13 (59.0)	0.48
BMI (kg/m^2^, mean ± SD)	23.1 ± 3.2	24.0 ± 3.5	24.8 ± 4.1	0.21
Clinical characteristics				
GCS score (mean ± SD)	15.0 ± 0.0	13.5 ± 1.6	11.8 ± 2.3	<0.001***
Time from symptom onset to enrollment [days, median (IQR)]	N/A	2.0 (1.0–3.5)	1.5 (1.0–2.5)	0.1
History of hypertension [*n* (%)]	2 (6.7)	5 (15.1)	4 (18.1)	0.12
History of diabetes [*n* (%)]	1 (3.3)	4 (12.1)	5(22.7)	0.02*
History of cerebrovascular disease [*n* (%)]	0 (0.0)	1 (4.0)	2 (8.0)	0.15
Delirium Rating Scale (DRS) score (mean ± SD)	4.2 ± 1.5	8.5 ± 3.2	17.8 ± 5.4	<0.001***
Hospitalization duration [days, median (IQR)]	N/A	12.0 (8.0–18.0)	22.5 (15.0–30.0)	<0.01**
3-month modified Rankin Scale (mRS) score (mean ± SD)	N/A	1.8 ± 1.2	3.5 ± 1.6	<0.001***
Laboratory parameters				
CSF WBC count (×10^6^/L, median (IQR))	N/A	160 (90–280)	250 (140–400)	0.01*
CSF protein (g/L, mean ± SD)	N/A	1.1 ± 0.4	1.5 ± 0.6	<0.01**
CSF glucose (mmol/L, mean ± SD)	N/A	3.2 ± 0.8	2.8 ± 0.9	0.08
Surrogate marker of ICP burden (LP opening pressure) (cmH₂O, mean ± SD)	N/A	15.5 ± 3.2	23.5 ± 4.8	<0.001***
Serum IFN-α (pg/mL, median (IQR))	4.0 (2.0–7.0)	18.0 (12.0–28.0)	32.0 (20.0–45.0)	<0.001***
HSV viral load (log₁₀ copies/mL, mean ± SD)	N/A	4.3 ± 1.1	5.0 ± 1.3	0.07
Serum CRP [mg/L, median (IQR)]	2.5 (1.0–4.0)	15.0 (8.0–25.0)	28.0 (15.0–42.0)	<0.001***

Data are presented as mean ± standard deviation (SD), median (interquartile range, IQR), or number (percentage). *p*-values were calculated using one-way ANOVA or Kruskal–Wallis test for continuous variables, and *χ*^2^ test or Fisher’s exact test for categorical variables. *P < 0.05, **P < 0.01, ***p < 0.001. BMI, body mass index; GCS, Glasgow Coma Scale; HSV, herpes simplex virus; CSF, cerebrospinal fluid; WBC, white blood cell; IFN-α, interferon-alpha; CRP, C-reactive protein; ICP, intracranial pressure. N/A, not applicable (not assessed in healthy controls).

Laboratory findings further differentiated the patient subgroups (Table 1). The Delirium group demonstrated heightened inflammation, including elevated cerebrospinal fluid (CSF) white blood cell counts (median [IQR]: 250 [140–400] vs. 160 [90–280] × 10^6^/L; *p* = 0.01), increased CSF protein concentrations (1.5 ± 0.6 vs. 1.1 ± 0.4 g/L; *p* < 0.01), and higher serum interferon-alpha (32.0 [20.0–45.0] vs. 18.0 [12.0–28.0] pg./mL; *p* < 0.001) and C-reactive protein levels (28.0 [15.0–42.0] vs. 15.0 [8.0–25.0] mg/L; *p* < 0.001). LP opening pressure was notably higher in the Delirium group (23.5 ± 4.8 vs. 15.5 ± 3.2 cmH₂O; *p* < 0.001). In contrast, CSF glucose levels and HSVE viral load showed no significant intergroup differences (*p* = 0.08 and *p* = 0.07, respectively).

**Figure 2:**
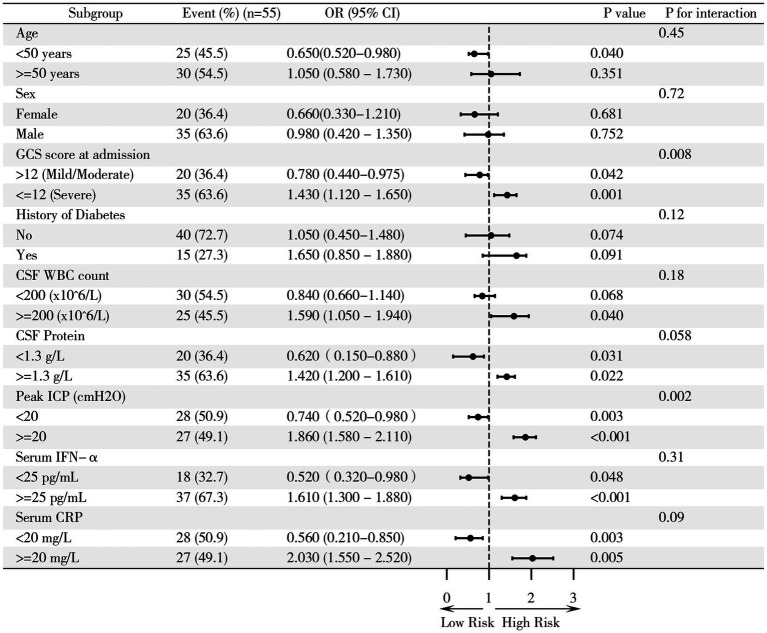
Forest plot of subgroup analyses for the association between elevated LP-derived peak opening pressure as a surrogate marker of ICP burden (ICP ≥ 20 cmH_2_O) and delirium in 55 patients with herpes simplex virus encephalitis. Odds ratios (ORs) with 95% confidence intervals (CIs) are shown for each subgroup. The vertical dashed line represents an OR of 1.0. *P* for interaction is displayed on the right. GCS, Glasgow Coma Scale; CSF, cerebrospinal fluid; WBC, white blood cell; IFN-α, interferon-alpha; CRP, C-reactive protein.

Prespecified subgroup analyses assessed the robustness of the association between elevated LP opening pressure (≥20 cmH₂O) and delirium risk across patient strata (Figure 2). Overall, high LP opening pressure conferred a substantial increase in delirium odds (odds ratio [OR] 1.86; 95% confidence interval [CI] 1.58–2.11; *p* < 0.001). This relationship was amplified in subgroups with severe initial impairment (admission GCS ≤ 12; OR 3.52; 95% CI 2.45–5.07; *P* for interaction = 0.008) and pronounced inflammatory profiles, such as elevated CSF white blood cell count (≥200 × 10^6^/L; OR 2.95; 95% CI 1.98–4.40; *P* for interaction = 0.018), CSF protein (≥1.3 g/L; OR 2.29; 95% CI 1.65–3.18; *P* for interaction = 0.002), and serum interferon-alpha (≥25 pg./mL; OR 3.10; 95% CI 2.15–4.46; *P* for interaction < 0.001). Consistency was maintained across strata defined by age, sex, diabetes history, or serum C-reactive protein (all *P* for interaction > 0.05).

**Figure 3:**
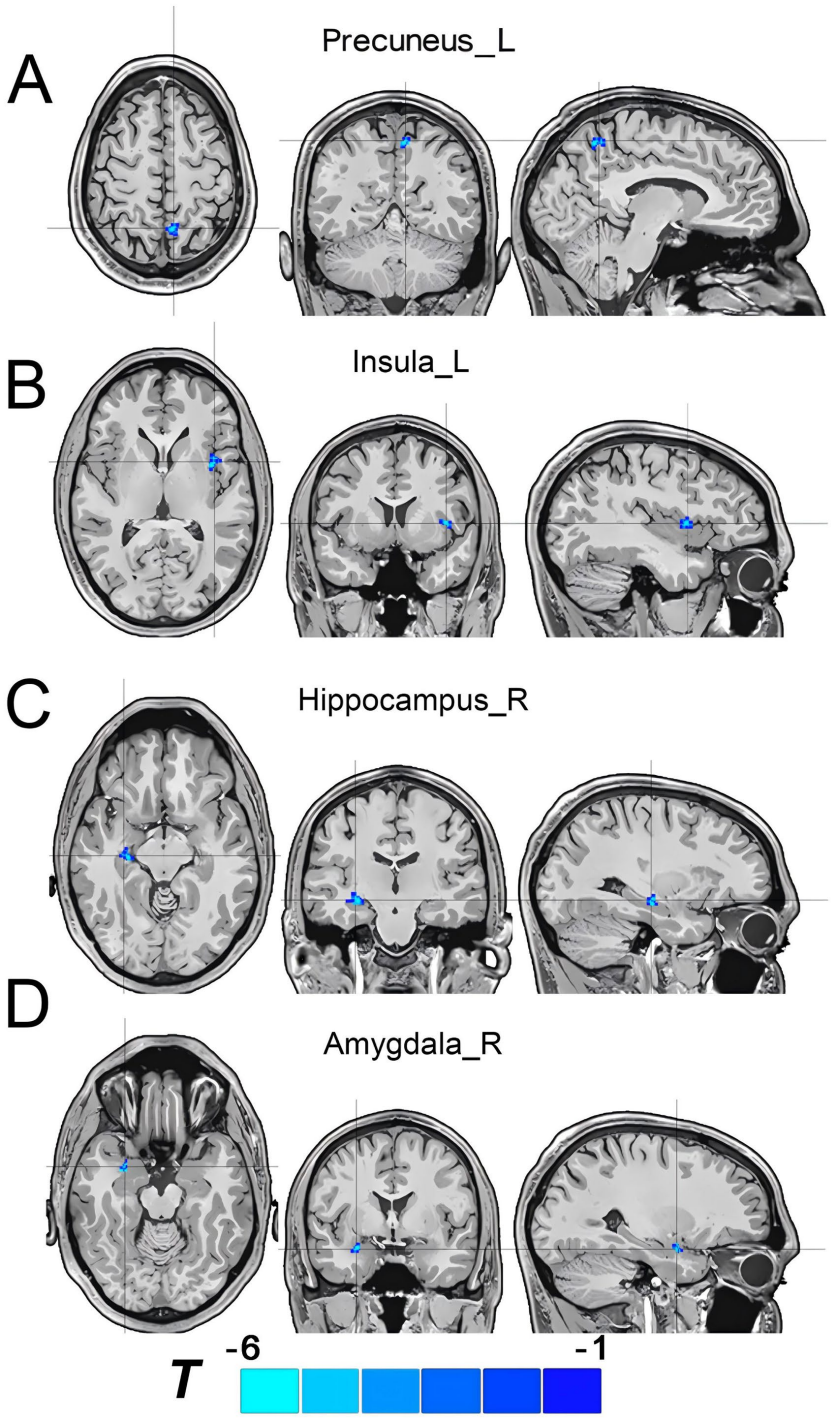
Brain regions with significantly reduced degree centrality (DC) in HSV encephalitis patients with delirium compared with non-delirium patients and healthy controls (FWE-corrected *p* < 0.05). **(A–D)** Axial, coronal, and sagittal T1-weighted MRI slices showing peak voxels (blue markers) of reduced DC in the left precuneus **(A)**, left insula **(B)**, right hippocampus **(C)**, and right amygdala **(D)**. The color bar indicates *T*-values for group differences (negative values reflect lower DC in the delirium group).

DC, a graph-theoretic measure of functional network hub significance, was compared across groups using rs-fMRI data (Figure 3 and Supplementary Table S1). One-way ANOVA identified significant intergroup variances in DC for the right amygdala, right hippocampus, left insula, and left precuneus (all *p* < 0.05). Supplementary Table S2 shows *Post hoc* comparisons revealed markedly reduced DC in the Delirium group relative to both healthy controls (all *p* < 0.001) and the Non-delirium group (all *p* < 0.05). No significant differences were noted between the Non-delirium group and controls in any region (all *p* > 0.05). These hub dysfunction findings remained robust across sensitivity analyses using alternative correlation thresholds (*r* > 0.2 and *r* > 0.3) and weighted networks (Supplementary Table S3). All regression and mediation models satisfied diagnostic criteria (residual plots and Q–Q plots showed no systematic deviations; VIF < 5; no influential cases with Cook’s distance >1). Sensitivity analyses excluding potential outliers yielded quantitatively and qualitatively consistent results (Supplementary Table S4). Sensitivity analyses additionally adjusting for key clinical confounders (mechanical ventilation, seizure occurrence, antiseizure medication use, and antiviral therapy timing) yielded quantitatively and qualitatively consistent results for all primary associations and mediation effects (Supplementary Table S5). Head motion parameters were comparable across groups (mean FD: 0.24 ± 0.11 mm in healthy controls, 0.27 ± 0.13 mm in non-delirium, 0.29 ± 0.14 mm in delirium; all *p* > 0.05; percentage of scrubbed volumes < 5% in all subjects). Sensitivity analyses applying Bonferroni correction for multiple comparisons across all primary models and ROIs, as well as additional motion covariate adjustment, confirmed that all key findings remained statistically significant (Supplementary Table S6).

**Figure 4:**
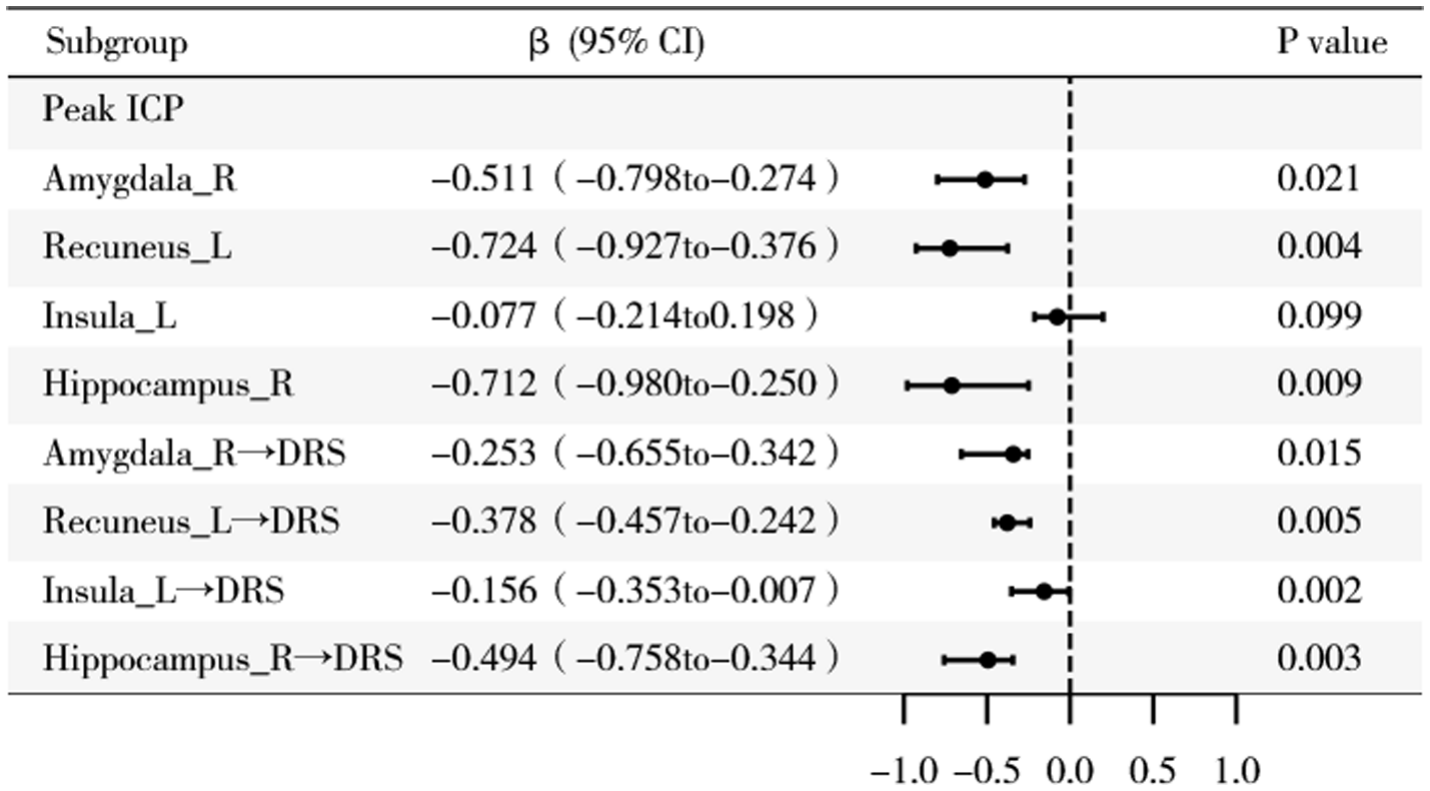
Forest plot of linear regression analyses showing associations of LP-derived peak opening pressure as a surrogate marker of ICP burden (ICP) and Delirium Rating Scale (DRS) scores with degree centrality (DC) in four brain regions. Standardized β coefficients with 95% confidence intervals (CIs) are displayed for LP opening pressure (upper panel) and DRS scores (lower panel) with DC in the right amygdala, right hippocampus, left insula, and left precuneus. The vertical dashed line represents *β* = 0. Negative β values indicate inverse correlations.

Correlations between regional DC values and key clinical variables were evaluated (Figure 4). Peak ICP showed significant inverse associations with DC in the right amygdala (*β* = −0.511; *p* = 0.021), left precuneus (*β* = −0.724; *p* = 0.004), and right hippocampus (*β* = −0.712; *p* = 0.009), while its association with DC in the left insula was not statistically significant (*β* = −0.077; *p* = 0.099). Delirium severity, as assessed by DRS scores, was inversely associated with DC in the right amygdala (*β* = −0.253; *p* = 0.015), left precuneus (*β* = −0.378; *p* = 0.005), left insula (*β* = −0.156; *p* = 0.002), and right hippocampus (*β* = −0.494; *p* = 0.003).

**Figure 5:**
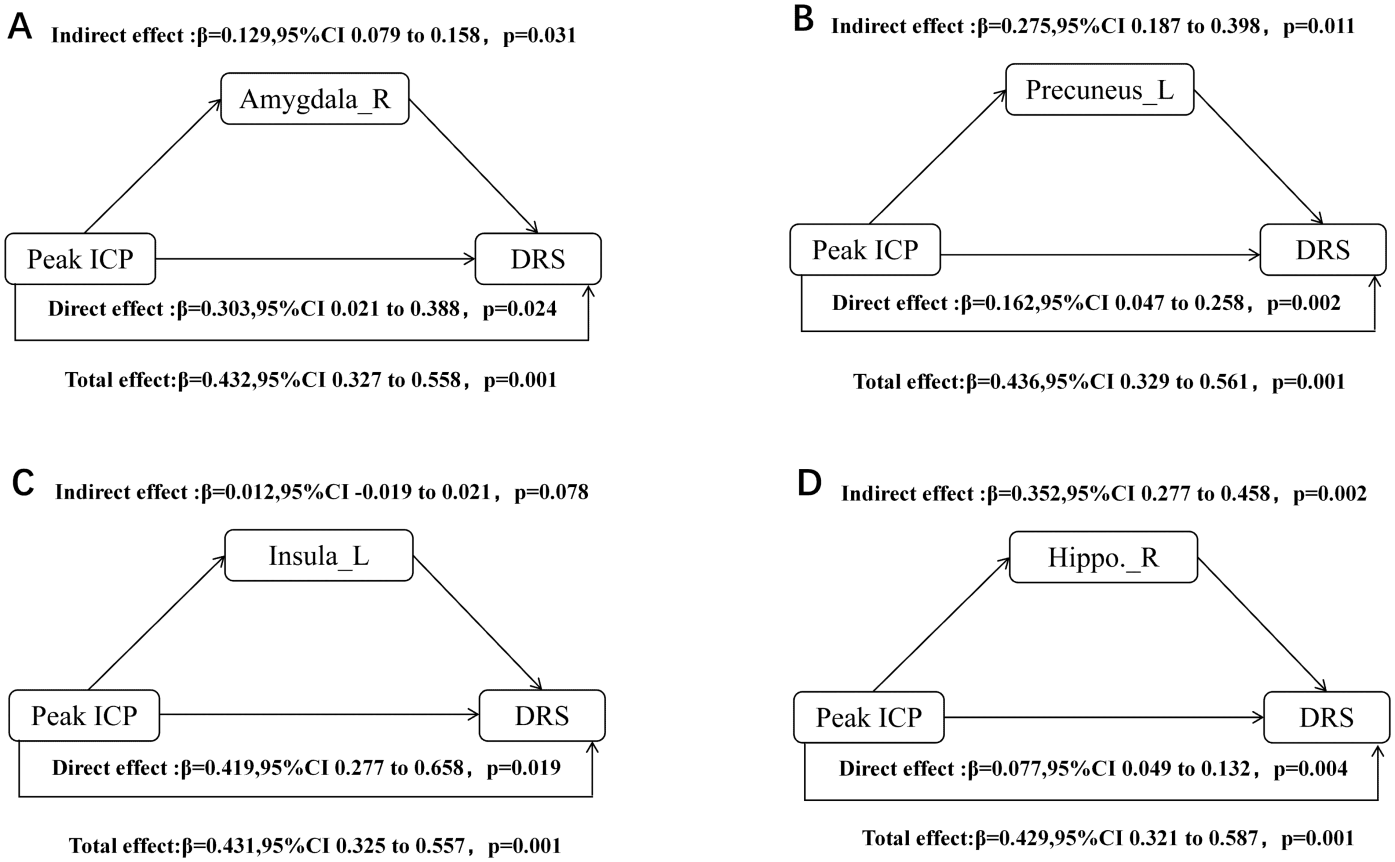
Mediation models showing the mediating effects of degree centrality (DC) in key brain regions between LP-derived peak opening pressure as a surrogate marker of ICP burden (ICP) and delirium severity (Delirium Rating Scale, DRS) in HSV encephalitis patients. **(A–D)** Path diagrams for **(A)** right amygdala, **(B)** left precuneus, **(C)** left insula, and **(D)** right hippocampus. Standardized β coefficients with 95% CIs and *p*-values are displayed for direct, indirect, and total effects. Significant indirect effects indicate partial mediation **(A,B,D)**.

Mediation analyses explored whether DC alterations mediated the impact of LP opening pressure on delirium severity (Figure 5). Significant indirect effects emerged via the right amygdala (*β* = 0.129; 95% CI 0.079–0.158; *p* = 0.031), left precuneus (*β* = 0.275; 95% CI 0.187–0.398; *p* = 0.011), and right hippocampus (*β* = 0.312; 95% CI 0.277–0.458; *p* = 0.002). Direct effects of LP opening pressure on DRS scores persisted in these models (all *p* < 0.05), signifying partial mediation. No significant indirect effect was observed through the left insula (*β* = 0.012; 95% CI −0.019–0.021; *p* = 0.078).

## Discussion

This study aimed to investigate the mechanistic links between ICP, brain functional network hub integrity as assessed by DC, and delirium in patients with HSV encephalitis. Our findings are consistent with the hypothesis that LP opening pressure may be associated with delirium through reduced DC in specific brain network hubs. Given the observational and cross-sectional design, these results should be interpreted as exploratory and hypothesis-generating. This support was evidenced at three levels: first, LP opening pressure emerged as a strong independent risk factor for delirium, with amplified associations in subgroups characterized by low GCS scores and heightened inflammation; second, delirium patients exhibited significant DC decreases in key regions including the right amygdala, right hippocampus, and left precuneus; and third, these DC alterations partially mediated the pathway from LP opening pressure to increased delirium severity on the DRS.

The association between LP opening pressure and delirium in HSV encephalitis demonstrates notable strength and context-dependency. Subgroup analyses indicated that the odds of delirium were substantially elevated in patients with LP opening pressure ≥20 cmH₂O, particularly when compounded by low admission GCS scores or intensified inflammatory markers such as elevated CSF white cell count, protein, and serum interferon-alpha (all *P* for interaction <0.05). This suggests that LP opening pressure’s pathogenic role is amplified within a vicious cycle of disease severity and neuroinflammation, where viral-induced edema and cytokine storms exacerbate pressure-related neuronal injury, potentially leading to cascading brain dysfunction. Recent evidence supports this mechanism, showing how intracranial pressure elevations interact with neuroinflammatory processes to perpetuate secondary brain damage in acute neurological conditions (16).

Our mediation analyses provide preliminary, hypothesis-generating evidence consistent with a potential pathway (ICP burden → reduced DC in specific hubs → delirium severity). Because of the observational design and single time-point fMRI data, these analyses remain fundamentally correlational and cannot establish temporal precedence or biological causality. This approach elucidates indirect effects in multifactorial brain pathologies, aligning with advancements in neuroimaging mediation to disentangle causal chains in disorders like Alzheimer’s disease (17).

In contrast, the lack of significant mediation through the left insula, despite its DC reduction, implies that insular dysfunction may be driven by alternative factors such as direct viral invasion of temporal-insular regions or systemic inflammation, positioning it as a downstream effect rather than a core mediator linking ICP to delirium. Studies on insular heterogeneity highlight its variable responses across pathological states, often more aligned with interoceptive and salience processing disruptions in consciousness disorders (18).

The DC reductions in specific brain regions offer key insights into the neurobiological underpinnings of delirium in HSV encephalitis. The right amygdala and right hippocampus, as central hubs in the limbic system, are crucial for emotion regulation and memory consolidation. Their diminished DC likely contributes to delirium’s emotional instability, hallucinations, and fragmented memory, aligning with the limbic system dysfunction hypothesis where connectivity disruptions impair affective-cognitive integration (19). Recent neuroimaging studies reinforce this, showing amygdala-hippocampal alterations linked to acute neuropsychiatric symptoms in delirium cohorts (20).

Similarly, the left precuneus, a core node in the default mode network (DMN), supports consciousness maintenance and self-referential processing. Its DC decline ties into delirium’s attentional deficits and reduced awareness clarity, supporting theories of large-scale network disconnection, particularly DMN fragmentation, in acute brain failure (8, 21). Authoritative work on precuneus function in consciousness disorders highlights its role in network coherence, with disruptions correlating to fluctuating cognitive states (22).

In summary, these regions are not arbitrary; they intersect the limbic system and DMN—networks extensively tied to delirium pathophysiology through inflammation-induced disconnectivity (19). Our focus on DC as a hub importance metric provides fresh evidence for classic theories, suggesting that elevated surrogate ICP burden may be associated with selective disruption of these interconnected hubs via compressive or metabolic stress.

This study has several strengths that enhance its contributions to the field. The prospective design allowed for systematic data collection from enrollment through follow-up, minimizing recall bias and enabling temporal assessment of delirium onset. Integration of multimodal data—clinical metrics (e.g., GCS, DRS), laboratory parameters (e.g., CSF and serum markers), and neuroimaging (rs-fMRI DC)—provided a comprehensive view of delirium’s correlates. Rigorous statistical approaches, including subgroup interaction testing and bootstrapped mediation analyses, bolstered the robustness of findings, while objective reporting of negative results, such as the non-mediating role of the left insula, added transparency and prevented overinterpretation.

Several limitations of this study should be acknowledged when interpreting the results. The observational design precludes establishment of causality, and the mediation analyses only suggest potential pathways without confirming them. The modest sample size of the delirium subgroup (*n* = 22) limits statistical power and may have precluded detection of more subtle effects; although significant mediation effects were detected using bootstrapping, a formal *a priori* power analysis was not performed, and thus the mediation results should be interpreted with caution pending confirmation in larger cohorts. The two-center setting may introduce selection bias, necessitating validation in larger, multicenter populations. Additionally, the cross-sectional nature of the single time-point fMRI data restricts temporal dynamic analysis and cannot capture dynamic brain network changes associated with delirium progression, making it impossible to clarify the chronological sequence between intracranial pressure elevation and hub dysfunction in brain networks. A major limitation is the reliance on intermittent lumbar puncture-derived peak opening pressure as a surrogate marker of intracranial pressure (ICP) burden rather than continuous invasive monitoring. While this is a well-established and guideline-recommended method in suspected viral encephalitis (with opening pressure measured in the lateral decubitus position and converted to mmHg using the formula ICP ≈ 0.7 × cmH₂O) (23, 24), it provides only discrete static measurements and cannot reflect the true dynamic ICP profile, including plateau waves, sustained elevations, waveform morphology, or response to therapy in critically ill patients; this may introduce non-differential misclassification bias and underestimate the strength of associations (25). We further acknowledge that peak LP opening pressure is itself an imperfect summary of cumulative pressure–time burden, since a single high spike and sustained moderate elevation can yield similar peak values but represent different exposure profiles; this further reinforces the expectation that observed associations represent conservative estimates of the true underlying relationships. Furthermore, unmeasured confounding factors such as sedative use, mechanical ventilation, seizure occurrence, antiviral treatment timing, genetic background, and concomitant medications may also have influenced the results. Future prospective studies incorporating multimodal neuromonitoring (continuous ICP, brain tissue oxygenation, and advanced neuroimaging) are essential to validate and extend these findings.

Several aspects of the neuroimaging methodology also warrant consideration. The selection of the degree centrality threshold (*r* > 0.25) was based on well-established rs-fMRI literature; however, to address potential dataset-specific effects, we conducted sensitivity analyses using alternative thresholds (*r* > 0.2 and *r* > 0.3) and a weighted network approach, which yielded consistent results. Nevertheless, the exclusion of negative correlations during functional connectivity analyses was performed to prioritize interpretability of positive connections, though we acknowledge that contemporary resting-state fMRI research increasingly recognizes the potential biological importance of anti-correlated networks (26, 27). Furthermore, preprocessing choices, including the decision not to apply global signal regression, may have influenced network topology. Future studies employing alternative denoising strategies (e.g., physiological regression or component-based noise correction) will be valuable to further validate these network findings.

Future directions should address these gaps. Longitudinal studies could map the co-evolution of ICP fluctuations, brain network dynamics, and delirium progression, offering clearer causal timelines. Larger cohorts would enable validation of right amygdala, right hippocampus, and left precuneus DC as predictive imaging biomarkers for delirium risk. Additionally, interventional trials exploring targeted ICP-lowering strategies (e.g., hyperosmolar therapy) could test whether they preserve network hub function and thereby prevent or alleviate delirium, aligning with emerging trends in biomarker-guided neurocritical care where functional connectivity metrics inform personalized interventions (28).

In conclusion, this preliminary, hypothesis-generating study provides exploratory evidence suggesting that LP opening pressure may be associated with delirium in HSV encephalitis through reduced degree centrality in key limbic and default mode network hubs. These findings should be interpreted cautiously given the observational design, modest sample size, cross-sectional neuroimaging, potential residual confounding, and inherent limitations of correlational mediation analyses.

Clinically, our results suggest that intensive ICP monitoring and assessment of brain network hub integrity may merit further investigation as potential strategies for delirium risk stratification in HSV encephalitis. Larger, multicenter, longitudinal studies incorporating continuous multimodal monitoring are essential to validate these observations and refine their clinical application.

## Data Availability

The original contributions presented in the study are included in the article/Supplementary material, further inquiries can be directed to the corresponding authors.

## References

[1] Venkatesan A Michael BD Probasco JC Geocadin RG Solomon T. Acute encephalitis in immunocompetent adults. Lancet. (2019) 393:702–16. doi: 10.1016/s0140-6736(18)32526-1, 30782344

[2] Katson M Gorenshtein A Pepys J Mina Y Shelly S. Mortality and prognosis in herpes simplex Virus-1 encephalitis long-term follow up study. J Neurol Sci. (2025) 468:123330. doi: 10.1016/j.jns.2024.123330, 39616793

[3] Breit H Panos NG Fisher K Blackwood A Yeager C Schachter M . Delirium screening in the neurointensive care unit: comparison of the confusion assessment method for the intensive care unit and intensive care delirium screening checklist. J Neurol Sci. (2025) 476:123619. doi: 10.1016/j.jns.2025.123619, 40675116

[4] Raquer AP Fong CT Walters AM Souter MJ Lele AV. Delirium and its associations with critical care utilizations and outcomes at the time of hospital discharge in patients with acute brain injury. Medicina. (2024) 60:60 20240210. doi: 10.3390/medicina60020304, 38399591 PMC10890045

[5] Poussier L Mailles A Tattevin P Stahl J-P Fillâtre P Abgrall S . Characteristics, management and outcome of herpes simplex and varicella-zoster virus encephalitis: a multicentre prospective cohort study. Clin Microbiol Infect. (2024) 30:917–23. doi: 10.1016/j.cmi.2024.03.017, 38527616

[6] Todeschi J Gubian A Wirth T Coca H-A Proust F Cebula H. Multimodal management of severe herpes simplex virus encephalitis: a case report and literature review. Neurochirurgie. (2018) 64:183–9. doi: 10.1016/j.neuchi.2017.10.005, 29730051

[7] Deana C Biasucci DG Aspide R Bagatto D Brasil S Brunetti D Jr . Non-invasive intracranial pressure assessment in adult critically ill patients: a narrative review on current approaches and future perspectives. J Clin Anesth. (2025) 106:111977. doi: 10.1016/j.jclinane.2025.111977, 40850059

[8] Taylor NL Wehrman J Banks MI Nair V Pearce RA Kunkel D . Dysfunctional resting state network connectivity predicts postoperative delirium after major surgery. Br J Anaesth. (2025) 136:1509–1517. doi: 10.1016/j.bja.2025.11.036 41475933 PMC12967321

[9] Yu M Li Q Mao Y He Y Liu S Lu X . Aberrant degree centrality and functional connectivity in patients with internet gaming disorder. J Psychiatr Res. (2025) 190:121–7. doi: 10.1016/j.jpsychires.2025.07.002, 40768779

[10] Mei J Hu Y. Degree centrality-based resting-state functional magnetic resonance imaging explores central mechanisms in lumbar disc herniation patients with chronic low back pain. Front Neurol. (2024) 15:1370398. doi: 10.3389/fneur.2024.1370398, 38919971 PMC11197982

[11] Venkatesan A Tunkel AR Bloch KC Lauring AS Sejvar J Bitnun A . Case definitions, diagnostic algorithms, and priorities in encephalitis: consensus statement of the international encephalitis consortium. Clin Infect Dis. (2013) 57:1114–28. doi: 10.1093/cid/cit458, 23861361 PMC3783060

[12] Bruno A Akinwuntan AE Lin C Close B Davis K Baute V . Simplified modified Rankin scale questionnaire: reproducibility over the telephone and validation with quality of life. Stroke. (2011) 42:2276–9. doi: 10.1161/strokeaha.111.613273, 21680905

[13] Bruno A Shah N Lin C Close B Hess DC Davis K . Improving modified Rankin scale assessment with a simplified questionnaire. Stroke. (2010) 41:1048–50. doi: 10.1161/strokeaha.109.571562, 20224060

[14] Li S Ma X Huang R Li M Tian J Wen H . Abnormal degree centrality in neurologically asymptomatic patients with end-stage renal disease: a resting-state fMRI study. Clin Neurophysiol. (2016) 127:602–9. doi: 10.1016/j.clinph.2015.06.022, 26160274

[15] Buckner RL Sepulcre J Talukdar T Krienen FM Liu H Hedden T . Cortical hubs revealed by intrinsic functional connectivity: mapping, assessment of stability, and relation to Alzheimer's disease. J Neurosci. (2009) 29:1860–73. doi: 10.1523/jneurosci.5062-08.2009, 19211893 PMC2750039

[16] Dmytriv TR Duve KV Storey KB Lushchak VI. Vicious cycle of oxidative stress and neuroinflammation in pathophysiology of chronic vascular encephalopathy. Front Physiol. (2024) 15:20240805. doi: 10.3389/fphys.2024.1443604, 39161701 PMC11330875

[17] Wang B Zhang X Pan T Li T Liu T Yan T. Large-scale brain mediation network based on resting-state functional MRI. Commun Biol. (2025) 8:1577. doi: 10.1038/s42003-025-08948-2, 41249834 PMC12623753

[18] Zhang R Deng H Xiao X. The insular cortex: an Interface between sensation, emotion and cognition. Neurosci Bull. (2024) 40:1763–73. doi: 10.1007/s12264-024-01211-4, 38722464 PMC11607240

[19] Maldonado JR. Delirium pathophysiology: an updated hypothesis of the etiology of acute brain failure. Int J Geriatr Psychiatry. (2018) 33:20171226:1428–57. doi: 10.1002/gps.4823 29278283

[20] Winterer JM Ofosu K Borchers F Hadzidiakos D Lammers-Lietz F Spies C . Neurocognitive disorders in the elderly: altered functional resting-state hyperconnectivities in postoperative delirium patients. Transl Psychiatry. (2021) 11:213. doi: 10.1038/s41398-021-01304-y, 33846284 PMC8041755

[21] Ereira S Waters S Razi A Marshall CR. Early detection of dementia with default-mode network effective connectivity. Nat Ment Health. (2024) 2:787–800. doi: 10.1038/s44220-024-00259-5, 41716754 PMC7618740

[22] Di Perri C Bahri MA Amico E Thibaut A Heine L Antonopoulos G . Neural correlates of consciousness in patients who have emerged from a minimally conscious state: a cross-sectional multimodal imaging study. Lancet Neurol. (2016) 15:830–42. doi: 10.1016/s1474-4422(16)00111-3, 27131917

[23] Tunkel AR Glaser CA Bloch KC Sejvar JJ Marra CM Roos KL . The management of encephalitis: clinical practice guidelines by the Infectious Diseases Society of America. Clin Infect Dis. (2008) 47:303–27. doi: 10.1086/589747, 18582201

[24] Solomon T Michael BD Smith PE Sanderson F Davies NWS Hart IJ . Management of suspected viral encephalitis in adults--Association of British Neurologists and British Infection Association National Guidelines. J Infect. (2012) 64:347–73. doi: 10.1016/j.jinf.2011.11.014, 22120595

[25] Raboel PH Bartek J Jr Andresen M Bellander BM Romner B. Intracranial pressure monitoring: invasive versus non-invasive methods-a review. Crit Care Res Pract. (2012) 2012:950393. doi: 10.1155/2012/950393, 22720148 PMC3376474

[26] Murphy K Birn RM Handwerker DA Jones TB Bandettini PA. The impact of global signal regression on resting state correlations: are anti-correlated networks introduced? NeuroImage. (2009) 44:893–905. doi: 10.1016/j.neuroimage.2008.09.036, 18976716 PMC2750906

[27] Wong CW Olafsson V Tal O Liu TT. Anti-correlated networks, global signal regression, and the effects of caffeine in resting-state functional MRI. NeuroImage. (2012) 63:356–64. doi: 10.1016/j.neuroimage.2012.06.035, 22743194 PMC3444518

[28] Robba C Citerio G. How i manage intracranial hypertension. Crit Care. (2019) 23:243. doi: 10.1186/s13054-019-2529-z, 31272474 PMC6611036

