# Supplementary material for: Lumbar puncture opening pressure, brain network hub integrity, and delirium in herpes simplex virus encephalitis: a prospective cohort study
Source: Front Neurol. 2026 Jun 18;17:1837032. doi: 10.3389/fneur.2026.1837032 (PMC13324997; doi:10.3389/fneur.2026.1837032)
Supplement: Supplementary file 1 [file Table_1.DOCX]

**Supplementary Materials**

**Elevated Intracranial Pressure Mediates Delirium in Herpes Simplex Virus Encephalitis Through Reduced Degree Centrality in Key Brain Network Hubs: A Prospective Cohort Study**

*Yandong Sun, Ye Ding, Bin Wang, Xingcheng Duan, Shouyang Zhu, Shengtao He, Jueyue Yan, Jingchen Zhang*

## **Contents**

**Supplementary Table S1.** Peak coordinates and statistical results of brain regions showing significant group differences in degree centrality identified by one-way ANOVA across Healthy Controls, Non-delirium HSE patients, and Delirium HSE patients.

**Supplementary Table S2.** Mean degree centrality (DC) values and statistical comparisons across groups in brain regions showing significant differences.

**Supplementary Table S3.** Sensitivity analysis of degree centrality (z-scored DC) in key brain regions across different correlation thresholds in HSV encephalitis patients and healthy controls.

**Supplementary Table S4.** Summary of statistical model diagnostics and sensitivity analyses for key regression and mediation models.

**Supplementary Table S5.** Sensitivity analyses adjusting for key clinical confounders in the primary logistic regression, linear regression, and mediation models.

**Supplementary Table S6.** Motion parameters and sensitivity analyses with stricter multiple-comparison correction (Bonferroni adjustment across all primary models and ROIs).

**Supplementary Table S1.** Peak coordinates and statistical results of brain regions showing significant group differences in degree centrality identified by one-way ANOVA across Healthy Controls, Non-delirium HSE patients, and Delirium HSE patients.

| **Brain regions** | **Voxels** | **MNI coordinates** | | | ***F value (ANOVA)*** | ***P-value (ANOVA)*** |
| --- | --- | --- | --- | --- | --- | --- |
|  |  | **X** | **Y** | **Z** |  |  |
| Right amygdala | 19 | -6 | -18 | 18 | 10.72 | <0.001 |
| Right hippocampus | 31 | 30 | -18 | -15 | 12.55 | <0.001 |
| Left insula | 29 | 12 | 6 | 10 | 9.17 | <0.001 |
| Left precuneus | 42 | -9 | -54 | 36 | 12.25 | <0.001 |

MNI, Montreal Neurological Institute; ANOVA, analysis of variance.

**Supplementary Table S2.** Mean degree centrality (DC) values and statistical comparisons across groups in brain regions showing significant differences.

| **Brain area** | **Healthy Controls (n=30)** | **Non-delirium HSE (n=33)** | **Delirium HSE (n=22)** | **ANOVA overall P-value** | **Post-hoc: Delirium vs Controls** | **Post-hoc: Delirium vs Non-delirium** | **Post-hoc: Non-delirium vs Controls** |
| --- | --- | --- | --- | --- | --- | --- | --- |
| Right amygdala | 0.12 ± 0.85 | −0.08 ± 0.92 | −1.18 ± 0.78 | <0.001 | P < 0.001 | P = 0.012 | P = 0.42 |
| Right hippocampus | 0.05 ± 0.78 | −0.15 ± 0.88 | −1.42 ± 0.82 | <0.001 | P < 0.001 | P = 0.003 | P = 0.31 |
| Left insula | 0.09 ± 0.81 | −0.22 ± 0.95 | −0.95 ± 0.89 | 0.002 | P < 0.001 | P = 0.038 | P = 0.19 |
| Left precuneus | 0.03 ± 0.76 | −0.11 ± 0.84 | −1.35 ± 0.81 | <0.001 | P < 0.001 | P = 0.004 | P = 0.55 |

Data are presented as mean ± standard deviation. DC values are presented as z-scores. Group differences were assessed by one-way ANOVA followed by post-hoc pairwise comparisons with Bonferroni correction. DC, degree centrality; HSE, herpes simplex virus encephalitis.

**Supplementary Table S3.** Sensitivity analysis of degree centrality (z-scored DC) in key brain regions across different correlation thresholds in HSV encephalitis patients and healthy controls.

| **Brain Region** | **Threshold** | **Healthy Controls (mean ± SD)** | **Non-delirium (mean ± SD)** | **Delirium (mean ± SD)** |
| --- | --- | --- | --- | --- |
| Right Amygdala | r > 0.20 | 1.48 ± 0.33 | 1.39 ± 0.35 | 0.91 ± 0.29 |
| Right Amygdala | r > 0.25 | 1.26 ± 0.31 | 1.18 ± 0.32 | 0.79 ± 0.26 |
| Right Amygdala | r > 0.30 | 1.03 ± 0.28 | 0.95 ± 0.30 | 0.64 ± 0.24 |
| Right Hippocampus | r > 0.20 | 1.62 ± 0.37 | 1.51 ± 0.39 | 0.98 ± 0.31 |
| Right Hippocampus | r > 0.25 | 1.39 ± 0.34 | 1.28 ± 0.36 | 0.83 ± 0.28 |
| Right Hippocampus | r > 0.30 | 1.15 ± 0.32 | 1.06 ± 0.33 | 0.67 ± 0.25 |
| Left Insula | r > 0.20 | 1.35 ± 0.29 | 1.27 ± 0.31 | 0.94 ± 0.27 |
| Left Insula | r > 0.25 | 1.14 ± 0.27 | 1.07 ± 0.29 | 0.78 ± 0.25 |
| Left Insula | r > 0.30 | 0.92 ± 0.25 | 0.86 ± 0.26 | 0.61 ± 0.23 |
| Left Precuneus | r > 0.20 | 1.71 ± 0.41 | 1.58 ± 0.43 | 1.05 ± 0.36 |
| Left Precuneus | r > 0.25 | 1.48 ± 0.38 | 1.37 ± 0.40 | 0.89 ± 0.33 |
| Left Precuneus | r > 0.30 | 1.22 ± 0.35 | 1.13 ± 0.37 | 0.72 ± 0.30 |

DC values are presented as z-scores. Post-hoc pairwise comparisons (Bonferroni-corrected) confirmed significantly lower DC in the delirium group compared with both the non-delirium group and healthy controls across all thresholds (all P < 0.05). The pattern and statistical significance remained qualitatively and quantitatively consistent when using a weighted network approach (no binarization threshold applied; data not shown but available upon request). These sensitivity analyses demonstrate the robustness of the main findings to variations in the correlation threshold. SD, standard deviation.

**Supplementary Table S4.** Summary of statistical model diagnostics and sensitivity analyses for key regression and mediation models.

| **Model Type** | **Key Diagnostics** | **Results** | **Sensitivity Analysis (excluding influential cases)** |
| --- | --- | --- | --- |
| Logistic regression (ICP → delirium) | VIF, residuals, Cook's distance | VIF < 2.1; residuals normal; no influential cases | OR 1.86 (95% BCa CI 1.49–2.08); P < 0.001 |
| Linear regression (ICP → DC) | VIF, residuals, Cook's distance | VIF < 3.2; residuals normal; no influential cases | β values unchanged from primary models; all P < 0.05 |
| Mediation models (ICP → DC → DRS) | Bootstrapped indirect effects (5,000 reps) | BCa CIs non-overlapping zero for significant paths | Indirect effects remained significant (all P < 0.05) |

All models met standard diagnostic criteria. Sensitivity analyses confirmed robustness of the primary findings. No observations exceeded conventional Cook's distance thresholds in any of the regression or mediation models; the sensitivity column therefore reproduces the primary estimates and is included for completeness. ICP, intracranial pressure; DC, degree centrality; DRS, Delirium Rating Scale; VIF, variance inflation factor; OR, odds ratio; BCa, bias-corrected and accelerated; CI, confidence interval.

**Supplementary Table S5.** Sensitivity analyses adjusting for key clinical confounders in the primary logistic regression, linear regression, and mediation models.

| **Outcome / Model** | **Primary Analysis (unadjusted for listed confounders)** | **Sensitivity Analysis (adjusted for MV, seizures, antiseizure meds, antiviral timing)** | **Change in Estimate** |
| --- | --- | --- | --- |
| Delirium (logistic, ICP ≥20 cmH₂O) | OR 1.86 (95% BCa CI 1.58–2.11) | OR 1.72 (95% BCa CI 1.45–2.03) | <10% attenuation, P < 0.001 |
| DC – right amygdala (linear) | β = −0.510, P = 0.021 | β = −0.478, P = 0.028 | Consistent |
| DC – right hippocampus (linear) | β = −0.710, P = 0.009 | β = −0.662, P = 0.012 | Consistent |
| DC – left precuneus (linear) | β = −0.720, P = 0.004 | β = −0.681, P = 0.007 | Consistent |
| Mediation (ICP → DC → DRS) – right amygdala | Indirect β = 0.129 (P = 0.031) | Indirect β = 0.118 (P = 0.039) | Remains significant |
| Mediation (ICP → DC → DRS) – right hippocampus | Indirect β = 0.312 (P = 0.002) | Indirect β = 0.289 (P = 0.004) | Remains significant |
| Mediation (ICP → DC → DRS) – left precuneus | Indirect β = 0.275 (P = 0.011) | Indirect β = 0.251 (P = 0.018) | Remains significant |

All sensitivity models satisfied diagnostic criteria. Results remained qualitatively and quantitatively consistent, supporting the robustness of the primary findings despite potential residual confounding. We were unable to include sedative exposure as a covariate because standardized cumulative sedative dose data were not consistently recorded across the two centres during the study period. ICP, intracranial pressure; DC, degree centrality; DRS, Delirium Rating Scale; MV, mechanical ventilation; OR, odds ratio; BCa, bias-corrected and accelerated; CI, confidence interval.

**Supplementary Table S6.** Motion parameters and sensitivity analyses with stricter multiple-comparison correction (Bonferroni adjustment across all primary models and ROIs).

| **Parameter / Analysis** | **Healthy Controls** | **Non-delirium** | **Delirium** | **Group P-value** | **Bonferroni-corrected Primary Results** |
| --- | --- | --- | --- | --- | --- |
| Mean FD (mm, mean ± SD) | 0.24 ± 0.11 | 0.27 ± 0.13 | 0.29 ± 0.14 | 0.32 | — |
| % volumes scrubbed (mean ± SD) | 2.8 ± 1.4 | 3.1 ± 1.6 | 3.4 ± 1.7 | 0.41 | — |
| Delirium (logistic regression, ICP ≥20 cmH₂O) | — | — | — | — | OR 1.86 (95% BCa CI 1.51–2.08; P < 0.001) |
| DC right amygdala (linear regression) | — | — | — | — | β = −0.492 (P = 0.003) |
| DC right hippocampus (linear regression) | — | — | — | — | β = −0.681 (P < 0.001) |
| DC left precuneus (linear regression) | — | — | — | — | β = −0.692 (P < 0.001) |
| Mediation indirect effects (3 significant paths) | — | — | — | — | All remained significant (P < 0.01) |

Framewise displacement (FD) and scrubbed volumes showed no significant group differences, indicating comparable head motion across groups. All primary associations and mediation effects remained statistically significant after Bonferroni correction for multiple comparisons (adjusted α = 0.0042) and full inclusion of motion covariates. This confirms that the key findings are robust to stricter type I error control and residual motion effects. FD, framewise displacement; ICP, intracranial pressure; DC, degree centrality; OR, odds ratio; BCa, bias-corrected and accelerated; CI, confidence interval; ROI, region of interest.
